# Immune challenge affects reproductive behaviour in the guppy (*Poecilia reticulata*)

**DOI:** 10.1098/rsos.230579

**Published:** 2023-08-09

**Authors:** Stella A. Encel, Emily K. Simpson, Timothy M. Schaerf, Ashley J. W. Ward

**Affiliations:** School of Life and Environmental Sciences, University of Sydney, Camperdown 2006, Australia

**Keywords:** reproduction, courtship, immunity, LPS, immunocompetence

## Abstract

Immunocompetence and reproduction are among the most important determinants of fitness. However, energetic and metabolic constraints create conflict between these two life-history traits. While many studies have explored the relationship between immune activity and reproductive fitness in birds and mammals inoculated with bacterial endotoxin, very few have focused on fish. Fish have been neglected in this area due, in part, to the claim that they are largely resistant to the immune effects of endotoxins. However, the present study suggests that they are susceptible to significant effects with respect to reproductive behaviour. Here, we examined the reproductive behaviour of male guppies following exposure to bacterial lipopolysaccharides (LPS) in comparison to that of male guppies in a control treatment. Additionally, we investigated the responses of females to these males. We show that although immune challenge does not suppress general activity in male guppies, it significantly reduces mating effort. While females showed no difference in general activity as a function of male treatments, they did exhibit reduced group cohesion in the presence of LPS-exposed males. We discuss this in the context of sickness behaviours, social avoidance of immune-challenged individuals and the effects of mounting an immune response on reproductive behaviour.

## 1. Introduction

Reproduction is fundamental to individual fitness, and among animals that reproduce sexually, courtship behaviour and mate choice are integral. As such, any factors which affect the courtship process may have significant repercussions for the lifetime reproductive output of an individual. In the case of animals which advertise mate quality with sexual ornaments and courtship behaviour, the quality of displays and sexual ornaments may be influenced by a number of factors including nutrition and immune activity [1]. Disease, and the ability to resist disease, are powerful agents of selection. As such, the transmission of alleles which confer immunocompetence to offspring may be the most essential consideration for any individual when assessing the quality of a potential mate [2]. Thus, the effects of immune activity on courtship and mating behaviour may have profound effects on an individual's reproductive success.

Mounting an immune response may exact significant metabolic and energetic costs [3,4]. Naturally, these costs detract from an individual's ability to devote resources to other functions such as growth and reproduction. As both immunocompetence and reproduction are essential components of fitness which are accompanied by significant physiological and energetic demands, the ability to manage the trade-off between these life-history traits may exert strong selection on individuals [3,5]. Additionally, cytokine-induced inflammatory immune responses cause a suite of behavioural changes known as sickness behaviours which may have a diminishing effect on an animal's general activity and social interaction [6,7]. The ubiquity of these inflammatory responses in the vertebrate immune system means that understanding the behavioural effects of these processes is of broad importance.

Animals mounting an immune response produce chemical and visual cues which indicate their infection status, often giving rise to social avoidance of the immune challenged individual [8–10]. While there is evidence to suggest that avoidance of diseased individuals can have powerful mitigating effects on the spread of disease [8,11,12], such avoidance may limit access to mating opportunities for the diseased individual. The negative effects of immune challenge on potential reproductive output [13,14] combined with those on perceived attractiveness [15–17] and social interaction [11,12] may significantly diminish the fitness of an affected individual. However, reduced attractiveness and access to mating opportunities may be compensated for through increased mating effort [18]. In light of this, it is important to pursue a comprehensive understanding of how immune activity may affect reproductive behaviour. Although much research has focused on the effects of disease and immune activation on traits such as mating coloration and sexual ornaments [16,19,20], less is known about how reproductive behaviour may be affected.

Several influential studies have examined the relationship between immune activity and reproductive traits in animals infected with whole live pathogens or parasites [2,16]. While this approach can offer valuable insights, the specificity of symptoms arising from a given disease may limit the ability to draw broad inferences across studies and taxa. Additionally, in the case of infectious diseases, it may be difficult to prevent transmission from infected to uninfected individuals tested or housed together [8]. For these reasons, the use of bacterial endotoxin (also known as Lipopolysaccharide; LPS) offers an ideal method for experimentally inducing inflammatory immune responses [21]. The utility of this method is evidenced by the widespread usage of LPS in both immunological and behavioural studies which seek to examine the effects of non-specific immune activity in vertebrates [22,23]. While there have been a few valuable studies which examine the sickness behaviours of fish more broadly [10,24], none of these have investigated the effects of immune challenge on the reproductive behaviour of fish. Here, we attempt to address this deficit in the existing literature.

In this study we examine the effects of immune challenge on the reproductive behaviour of male guppies (*Poecilia reticulata*). Guppies have a promiscuous mating system in which males devote a high proportion of their time and energy to courtship and mating [25]. Male guppies exhibit stereotypical courtship and mating behaviours which are characterized by sigmoidal displays and gonopodial thrusts, both of which are readily identifiable to an observer [25]. We induced immune challenge using bacterial endotoxin from *Escherichia coli*, which is a well-established means of experimentally stimulating an inflammatory immune response in vertebrates.

## 2. Material and methods

### 2.1. Study species

The Trinidadian guppy (*Poecilia reticulata*) is a widely used model organism in ecology, behaviour and physiology studies [25], and increasingly they have been used in the context of disease ecology [26,27]. They are ideal subjects for the study of reproductive behaviour due to their clear sexual dimorphism and stereotypical courtship displays. Fish used in the study were adults, females measuring 26.6 ± 1.4 mm (mean ± standard deviation) and males, which expressed adult coloration and had fully formed gonopodia, measuring 17.3 ± 1.2 mm (mean ± standard deviation). The body lengths of the fish were measured using stills taken from the videos collected during the trials. Fish were the progeny of wild stock collected from feral populations in the Northern Territory (Australia). Fish used in the experiments were housed in 90L aquaria where they were kept at 25°C on a 12:12 light:dark cycle. Fish were fed daily ad libitum with commercially available fish flakes (Nutrafin).

### 2.2. Immune challenge

Males were randomly assigned to either LPS or control treatments (LPS-exposed: *N* = 16, Control: *N* = 16). Groups of 8 males were placed in a 500 ml bath of either LPS solution or plain aged tap water and left for 60 min before being transferred to aquaria. Fish assigned to the LPS treatment were bathed in a solution of aged tap water and LPS at a concentration of 100 mg l^−1^ (Sigma-Aldrich; Lipopolysaccharides from *Escherichia coli* serotype O111 : B4). This concentration was selected on the basis of previous studies which induced immune reactions in fish using similar dosages [28,29]. Following treatment, fish were monitored for signs of stress for 48 h prior to experiments. A 48-h interval between exposure and testing was chosen on the basis of precedent from previous published studies which show that the immune response peaks 48–168 h post-injection in a closely related warm water species (*Gambusia holbrooki*) [30]. The bath method was chosen over an intraperitoneal or intramuscular injection due to the small size of the males and the success of this method in other trials using small teleosts [29,31].

### 2.3. Testing protocols

At 48 hrs post-exposure fish were transferred from their individual holding tanks to a circular test arena with a diameter of 70 cm and a depth of 5 cm. Arenas were lit using cool white LED strips (6500 K) and surrounded with white screens to minimize external disturbance. All trials were filmed using a Canon G1X camera positioned 1.1 m above the arena at a frame rate of 24fps and a resolution of 1920p. Each trial was filmed for 12 min from the time of introduction. Following this, fish were removed and transferred to new holding tanks. Each fish was only used once. The males and females were taken from separate holding aquaria and were unfamiliar with each other.

We conducted two treatments, each involving test groups of 3 females and 1 male to a total of 4 fish per group. We selected this sex ratio on the basis of female-skewed sex ratios of guppies in the wild [32,33]. The treatments were LPS, in which the male guppy was LPS-exposed and control, in which the male was bathed in plain water. At the time of testing, one control male showed signs of ill health and was excluded on this basis. Hence, we performed (LPS-exposed: *N* = 16, Control: *N* = 15) trials to a total sample size of (*N* = 31).

### 2.4. Data extraction and analysis

For each trial, we extracted the last 10 min of video footage for analysis. This allowed the fish an initial 2 min acclimation period prior to data collection [34]. Videos were tracked using TRex [35]. XY coordinates for each fish over the 10 min test period were extracted. From these, we calculated individual speed and the mean distance between the fish within each trial. Furthermore, the number of displays and the number of mating attempts made by each focal male were counted. This process was performed blind by an observer who was not aware of the treatment. Both the displays of male guppies and their mating attempts are stereotypical and unmistakable to an observer. Displays involve the male approaching a female and then assuming a sigmoid body attitude, with fins fully spread, and quivering [36,37]. Mating attempts involve the male approaching the female from the rear and accelerating toward her in an attempt to locate her genital pore with his gonopodium, which involves the two fish being in contact briefly.

### 2.5. Analysis

Statistical analysis was performed using R [38], using the packages MASS [39] and effectsize [40], while figures were produced using ggplot2 [41]. Data were examined visually using QQ-plots and histograms. Subsequently, Shapiro-Wilk tests and Bartlett's tests were used to examine the assumptions of normality and homoscedasticity respectively. We ascertained that the assumptions of normality and homoscedasticity were met for mean male size, mean female size, male swimming speed, the mean distance of males to females and the mean distance between females. We analysed these using linear models, with treatment as the independent variable. The number of displays and of mating attempts were both count variables and were overdispersed. Consequently, we analysed these generalized linear models with a negative binomial error distribution, using the glm.nb from the MASS package. Finally, mean female swimming speed was non-normal, and right skewed. Using the model.sel feature from the package MuMIn [42] we established that a generalized linear model using a gamma distribution was best suited to the analysis. We report unstandardized *β* coefficients and their 95% confidence intervals throughout.

To create encounter frequency heatplots, we examined relative positioning within groups via graphs that illustrated the relative frequencies that individual females and males occupied given (x, y) coordinates relative to other group members following the method in section S1.5 of the electronic supplementary material of [43].

## 3. Results

There was no difference in male (*β* coefficient [95% CI]: 0.02 [−0.73, 0.77], *t*_1,29_ = 0.058, *p* = 0.955) or female (*β* coefficient [95% CI]: −0.07 [−0.82, 0.68], *t*_1,29_ = −0.188, *p* = 0.853) size between treatments.

**Figure 1.**
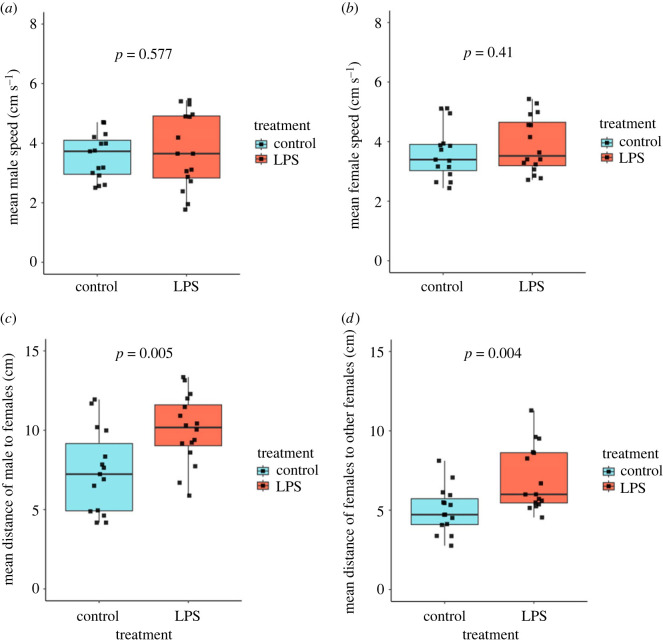
Box and whisker plots showing (*a*) the mean speed (cm s^−1^) of male guppies, (*b*) the mean speed (cm s^−1^) of female guppies, (*c*) the mean distance between a focal male and three females, and (*d*) the mean distance between females in two treatments (LPS-exposed or control). Data points are indicated by black squares, and the *p*-value resulting from the statistical comparison of the two treatments is shown at the top of each plot. While there was no difference in the mean swimming speeds of males or females between treatments, males in the LPS-exposed treatment were located at a significantly greater distance from females than in the control treatment, and females were located at significantly greater distances from other females in the treatment with LPS-exposed males than in the control treatment.

Although there was no significant difference in swimming speed between LPS-exposed and control males (*β* coefficient [95% CI]: 0.21 [−0.54, 0.95], *t*_1,29_ = 0.564, *p* = 0.577; figure 1*a*), LPS-exposed males were located at significantly greater mean distances from females than control males (*β* coefficient [95% CI]: 0.97 [0.32, 1.62], *t*_1,29_ = 3.056, *p* = 0.005; figure 1*c*). Similarly, there was also no difference in the mean swimming speed of females between the control and LPS treatments (*β* coefficient [95% CI]: 0.30 [−0.44, 1.04], *t*_1,29_ = 0.836, *p* = 0.41; figure 1*b*), however there was a significant difference between the treatments in regard to the distance between females (*β* coefficient [95% CI]: 0.98 [0.33, 1.63], *t*_1,29_ = 3.101, *p* = 0.004; figure 1*d*).

**Figure 2.**
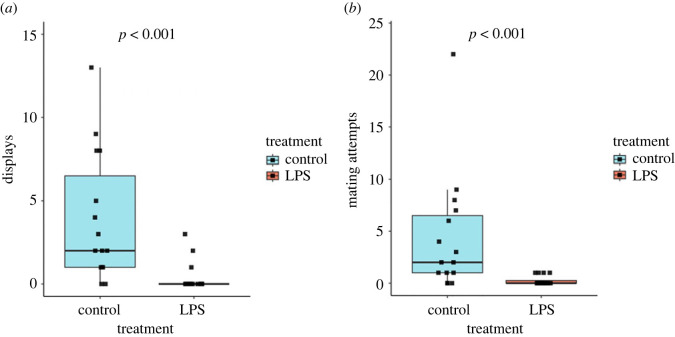
Box and whisker plot showing (*a*) the number of sexual displays made by male guppies, and (*b*) the number of mating attempts made by male guppies in two treatments (LPS-exposed or control). Data points are indicated by black squares, and the *p*-value resulting from the statistical comparison of the two treatments is shown at the top of each plot. LPS-treated males performed significantly fewer displays and made fewer mating attempts than control males.

LPS-exposed males also performed significantly fewer displays than control males (*β* coefficient [95% CI]: −2.35 [−3.50, −1.33], *Z*_1,29_ = −4.298, *p* < 0.001; figure 2*a*) and made significantly fewer mating attempts (*β* coefficient [95% CI]: −2.87 [−4.25, −1.69], *Z*_1,29_ = −4.468, *p* < 0.001; figure 2*b*).

**Figure 3.**
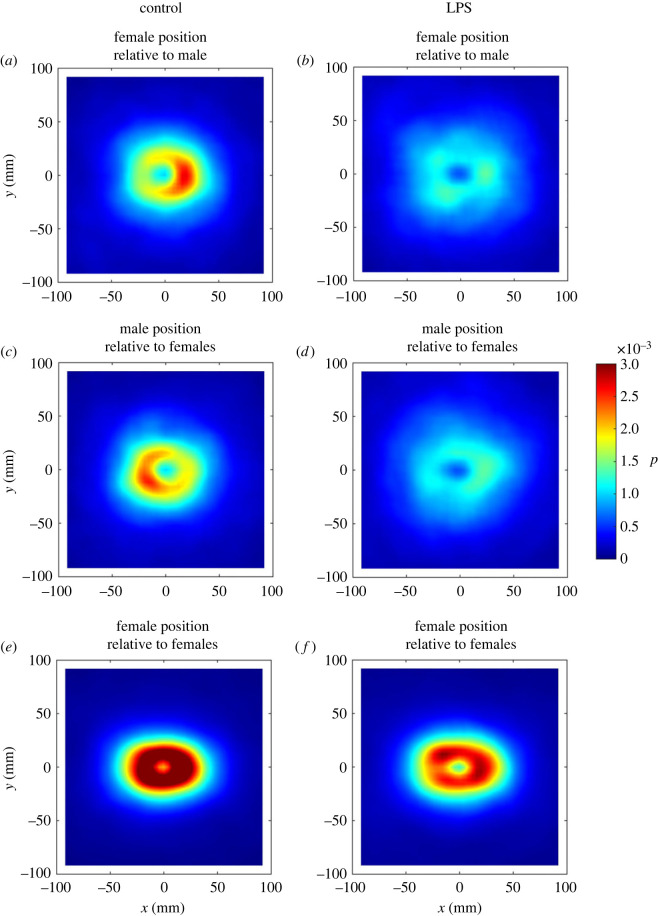
Plots showing the relative frequency, p, that females occupied given (x, y) coordinates relative to the position and direction of motion of their male group mates ((*a*) and (*b*)); that males occupied (x, y) coordinates relative to the positions and directions of motion of their female group mates ((*c*) and (*d*)); and, that females occupied (x, y) coordinates relative to the positions and directions of motion of their other female group mates ((*e*) and (*f*)), for control trials (left column, (*a*), (*c*) and (*e*)) and trials with LPS-exposed males (right column, (*b*), (*d*) and (*f*)). For each plot, the reference individual (the individual that the positions are relative to), is located at the origin (0, 0), with their direction of motion parallel to the positive x-axis (moving to the right).

The symmetry of panels (a) and (c) in figure 3 suggests that in control groups, the relative positioning of males and females was such that both males and females were moving in similar directions, with males positioned behind females, most frequently at distances of approximately 25 mm. This clear pattern vanished for LPS-treated males, who were positioned close to females at lower relative frequencies (figure 3*b,d*). Females tended to be slightly more densely packed relative to each other in control groups than in the presence of LPS-treated males (figure 3*e*,*f*).

## 4. Discussion

Sickness behaviours arising from inflammatory processes are reasonably consistent across vertebrate taxa, and typically involve reduced levels of activity and social interaction [10]. However, in the present study, we did not find any evidence for a reduction in general activity (measured as swimming speed). This finding has some precedent in previous work with immune-challenged fish which suggests they may be able to compensate partially for the metabolic costs of inflammation [30,44]. Despite this, there was a clear reduction in courtship and mating behaviour of immune-challenged males. This was accompanied by an increase in the distance between those males and the females in their groups. This may suggest that females avoid LPS-exposed males based on their perception of behavioural and/or chemical cues which indicate heightened immune activity in the male. Additionally, LPS-exposed males may suffer from a reduced motivation or ability to perform reproductive behaviours as a result of heightened immune activity. Moreover, although there was no difference in the swimming speed of females between treatments, females showed more cohesive shoaling in the control treatment. Previous literature suggests that female guppies (and other related fish) aggregate as a response to harassment by males attempting to mate with them [45–47]. Taken together, the findings here may reflect the need for both males and females to navigate a life-history trade-off between the considerations of survival and reproduction, both of which form essential components of fitness and which may conflict with one another.

Avoidance of diseased individuals is a key component of what has been referred to as the behavioural immune system [8,48], whereby healthy individuals mitigate their risk of infection by distancing themselves from conspecifics that are exhibiting symptoms of disease and/or heightened immune activity. This type of avoidance has been documented in a variety of species [11,17,48,49], including the guppy [26,27,44]. While the ability to detect and avoid individuals carrying transmissible diseases is generally desirable, it may be particularly important in the context of mate choice. When assessing prospective mates, the infection status of males is a crucial consideration for females as it reflects both the immediate risk of infection to herself [50] as well as the potential for males to contribute alleles which promote pathogen resistance in offspring. Research has shown that females of other species may respond aversively to olfactory and visual cues which indicate disease in prospective mates [15,17]. Here, females were located at greater distances from immune-challenged males, which may reflect female avoidance of such males. Furthermore, the differences in male behaviour between treatments may have influenced the positioning of females through a different mechanism. Specifically, the greater cohesion of females in the control treatment may be a result of the increased frequency of mating attempts by control males. This is concordant with existing literature which has shown that female aggregation may be a strategy to militate against male mating attempts [45,47,51].

Heightened immune activity in males involves a metabolic cost which likely limits their ability to express high energy behaviour patterns, such as those used in courtship displays [4]. This being the case, it would naturally be more difficult for immune-challenged males to mount courtship displays or indeed to attempt mating, particularly with avoidant females. Although there is evidence that individuals are able to suppress or modulate the expression of sickness behaviours in certain contexts [52], it may not always be advantageous for them to do so. In the case of a strong immune reaction which places especially high physiological demands on an animal, investment in sickness behaviours may be crucial in conserving resources necessary for survival [7]. This being the case, an individual may forgo other costly fitness related opportunities, in particular reproduction, to improve their chances of recovery and thus their ability to engage in future reproduction [53]. The idea that animals must balance investment in current and future reproduction is well-established in discussions of life-history traits, and is a component of the Pace of Life syndrome [54,55]. This framework could explain why immune-challenged males in this study demonstrate reduced mating effort. From the female perspective, males with low immunocompetence may be less likely to contribute to the production of high-quality offspring. As such, the costs of reproducing with these males may outweigh the benefits, which could explain why females in this study appear to actively avoid immune-challenged males. Distribution patterns revealed in the heatplots suggest that in the control treatment, males were most likely to be found in the region immediately behind the females, and to a lesser extent directly in front of them. This pattern is consistent with the typical mating strategy of male guppies; approaching females from behind while also occasionally performing displays in front of them [25]. Since neither male nor female swimming speed differed between treatments, the most parsimonious explanation for this trend is the diminished mating effort exhibited by immune-challenged males.

The Hamilton-Zuk hypothesis on the evolution of sexual ornamentation posits that displays may act as an honest indicator of pathogen resistance and genetic quality [19]. This means that immune function may have a powerful effect on the perceived attractiveness of an individual to prospective mates, with obvious ramifications for their reproductive success [16,19]. Several studies which have examined the relationship between male display traits and immune activity in vertebrates have suggested that the reduced reproductive fitness of immune-challenged males is underpinned by a reduction in circulating plasma testosterone [15,19,56]. The immunocompetence handicap hypothesis asserts that testosterone functions dually as a key component of sexual signal production in males and an immunosuppressant, creating a trade-off between immunity and reproduction [57]. While this hypothesis is speculative in nature and requires extensive further investigation, it could mean that only males carrying genes which confer high levels of pathogen resistance are able to withstand the immunosuppressive effects of the elevated testosterone levels needed to produce strong sexual signals. Although findings on the generalized immunosuppressive effects of testosterone are mixed [58,59], there appears to be some support for the related idea that heightened immune activity significantly suppresses levels of circulating testosterone [56]. If this is indeed true, it presents one possible mechanism for the results of this study; immune-challenged males may suffer from a limited ability to produce sexual signals and exert mating effort as a result of reduced testosterone levels. However, this requires further testing. While studies exploring the relationship between immune activity and reproduction have largely been conducted using birds and mammals, relatively few have focused on fish.

Fish are closely associated with gram-negative bacteria such as *E. coli* due to the ubiquity of such pathogens in waterways, and it has been stated that they are resistant to endotoxic shock as a result of this association [21]. However, it is also well-established that exposure to LPS provokes an array of immunological and physiological responses in fish [28,60]. Nonetheless, it has been claimed in some sources that fish do not respond behaviourally to the heightened immune activity associated with this exposure [21], despite the existence of longstanding evidence that fish manifest behavioural fever in response to infection with other gram-negative bacteria [61]. Furthermore, the clear differences in reproductive behaviour between LPS-exposed and control male guppies found in this study seem to suggest that fish may be subject to significant changes in behaviour which have simply not been investigated [10]. While generalized sickness behaviours such as lethargy may manifest differently in fish than in other vertebrates such as mammals and birds, they are not exempt from behavioural effects [10]. Rather, it may be that the behavioural effects of immune challenge with LPS in fish are more subtle or reveal themselves more readily with close examination of specific behaviours, as shown here. As such, our understanding in this area may be significantly improved by increasing the taxonomic diversity of experimental subjects to include more fish and other vertebrate taxa as well as birds and mammals.

Here, we show that an immune challenge significantly affects reproductive behaviour in male guppies, reducing the number of mating attempts and displays. Furthermore, we show that female guppies in the presence of immune-challenged males exhibit shoaling behaviour which is indicative of both female avoidance and reduced harassment from males. Valuable future work might identify the nature of cues involved in communicating immune status, and examine whether mating with an immune-challenged male carries a cost in terms of offspring number, size, or viability. It would also be of interest to examine if control males exert lower mating effort in the presence of immune-challenged females, or if they prefer to mate with control females.

## Ethics

All experiments were conducted with the approval of the University of Sydney Animal Ethics Committee (ref: 2022/2233).

## Data Availability

Data have been supplied as electronic supplementary material and will also be available on Dryad (doi:10.5061/dryad.866t1g1w7) [62].
